# Repression of GPRC5A is associated with activated STAT3, which contributes to tumor progression of head and neck squamous cell carcinoma

**DOI:** 10.1186/s12935-017-0406-x

**Published:** 2017-03-02

**Authors:** Shuli Liu, Dongxia Ye, Tong Wang, Wenzheng Guo, Hongyong Song, Yueling Liao, Dongliang Xu, Hanguang Zhu, Zhiyuan Zhang, Jiong Deng

**Affiliations:** 1 Department of Oral and Maxillofacial-Head and Neck Oncology, Ninth People’s Hospital, Shanghai Jiao Tong University School of Medicine, Shanghai, China; 2 Key laboratory of cell differentiation and apoptosis of Chinese Minister of Education, Shanghai Jiao Tong University School of Medicine, Shanghai, China; 3 Shanghai Key Laboratory for Tumor Microenvironment and Inflammation, Department of Biochemistry and Molecular Cell Biology, Shanghai Jiao Tong University School of Medicine, Shanghai, China

**Keywords:** GPRC5A, STAT3, Prognostic marker, Leukoplakia, HNSCC

## Abstract

**Background:**

G protein–coupled receptor family C group 5 member A (GPRC5A), a retinoic acid-inducible gene, is a lung tumor suppressor. Previously, we showed that repression of GPRC5A expression was associated with pathologic differentiation grade of oral squamous cell carcinomas (OSCC) and overexpression of GPRC5A gene inhibited the malignant phenotype in OSCC cells, suggesting that GPRC5A also functions as a tumor suppressor in oral cancer. However, the molecular mechanisms underlying GPRC5A deficiency in head and neck squamous cell carcinoma (HNSCC) are still unclear.

**Methods:**

In this study, we used Western blot analysis and immunohistochemical (IHC) staining to investigate the expression of GPRC5A in both HNSCC cell lines and clinical samples. GPRC5A stable transfectants and their parental HNSCC cells were characterized for their biological activities in anchorage-independent growth.

**Results:**

IHC analysis showed that, GPRC5A expression was high in normal tissue, but gradually decreased in oral leukoplakia, a precancerous stage, and greatly suppressed in primary cancer. Repression of GPRC5A was correlated with activated STAT3, which associates with aggressive clinicopathological features in HNSCC patients. Moreover, overexpression of GPRC5A suppressed IL-6-induced-STAT3 activation and inhibited anchorage-independent growth in HNSCC cells.

**Conclusions:**

Repressed GPRC5A associates with increased tumor grade and activated STAT3, which may be used as a prognostic marker for tumor progression of HNSCC.

## Background

Head and neck cancer is the sixth most frequent cancer worldwide and the 5-year survival rate is amongst the lowest of the major cancers [1]. More than 90% of head and neck cancer occurs in the oral cavity,and more than 90% of all malignant epithelial tumors arising in the oral cavity are squamous cell carcinomas [2, 3]. The concept that most oral squamous cell carcinomas (OSCC) arise from clinical pre-malignant lesions has been widely accepted [4]. Oral leukoplakia is one of the most frequent potentially malignant lesions, and about half of OSCC were associated with leukoplakia [5]. Therefore, understanding the molecular mechanisms of oncogenesis from precancerous lesions to malignant tumor progression may lead to revealing the mechanism of oncogenesis in head and neck squamous cell carcinoma (HNSCC), but also identification of novel molecular predictors for improvement of the prognosis.

G-protein coupled receptor family C group 5 member A (GPRC5A), a retinoic acid-inducible gene, was first cloned in 1998. GPRC5A is preferentially expressed in lung tissue, suggesting it may play an important role in this tissue. *Gprc5a* gene knockout lead to develop spontaneous lung tumors, indicating *Gprc5a* function as a lung tumor suppressor [6]. GPRC5A gene expression was frequently suppressed in lung cancer and HNSCC cells [7]. Previously, we showed that GPRC5A is expressed in normal oral tissue at relatively high level, whereas its expression was repressed in OSCC [8]. However, it is unknown whether GPRC5A expression is repressed in precancerous lesions, and how repression of GPRC5A is involved in the early stage of oncogenesis of HNSCC.

Intriguingly, persistent activation of STAT3 has been linked to tumorigenesis of HNSCC [9, 10]. STAT3 signaling can be triggered by cytokines and growth factors that regulate cell proliferation, differentiation, survival, invasion, inflammation and immunity [11]. Interleukin-6 (IL-6) and related cytokines bind to specific cell surface receptors, induce STAT activation via tyrosine phosphorylation at Y705 via the janus kinase (JAK) family kinases. The activated STAT proteins, as homo- or heterodimer, are then translocated into the nucleus to regulate gene transcription [11, 12]. Previously, STAT3 was found to be persistently activated in *Gprc5a* ^−/−^ mouse tracheal epithelial cells [13]. However, it is still unknown whether dysregulated STAT3 activation is resulted from GPRC5A deficiency and whether it is involved in tumorigenesis of HNSCC.

In this study, we examined GPRC5A expression in normal tissue, leukoplakia and primary tumors. We found that repression of GPRC5A occurred at an early stage of tumorigenesis, and associated with tumor progression. Moreover, we found that repression of GPRC5A is associated with activated STAT3 in HNSCC, which correlates with tumor progression. Importantly, overexpression of ectopic GPRC5A in HNSCC cells inhibits IL-6-induced STAT3 activation and suppresses the anchorage-independent tumor growth. Thus, repression of GPRC5A has been linked to upregulation of STAT3 signaling pathway in HNSCC, which contributes to tumor progression.

## Methods

### Patients and clinical samples

Forty oral leukoplakia samples and 86 paired HNSCC and adjacent normal tissues were obtained from the Ninth People’s Hospital (Shanghai, China). All samples were formalin-fixed, and paraffin-embedded from surgically resected specimens. Data were collected from clinical and pathologic records after approval by the Ethics Committee of the Ninth People’s Hospital of Shanghai.

### Reagents and antibodies

Mouse monoclonal antibody to the C-terminus of GPRC5A which was developed and purified in Abmart (Shanghai, China) and rabbit anti-GPRC5A polyclonal antibody were the same as described in our previous study [8]. Mouse anti-Actin monoclonal antibody was purchased from Santa Cruz Biotechnology (Santa Cruz, CA). Rabbit anti-phospho-STAT3 (Tyr705) monoclonal antibody, Mouse anti-STAT3 (124H6) monoclonal antibody and Rabbit anti-SOCS3 polyclonal antibody was purchased from Cell Signaling Technology (Beverley, MA). IL-6 was purchased from Santa Cruz Biotechnology (Santa Cruz, CA).

### Immunohistochemistry

The standard IHC method was used as previous reported [8]. Briefly, 3-μm sections were dewaxed in xylene and hydrated with graded ethanol. Then, antigen retrieval was carried out using 0.01 M citrate buffer (pH 6.0) pressure-cooking, and endogenous peroxidase activity was blocked with 3% hydrogen peroxide for 10 min at room temperature. After that, the sections were blocked for 1 h at room temperature with 5% normal goat serum. The slides were incubated with the primary antibody (1:150 for GPRC5A and 1:400 for p-STAT3) in a moist chamber for overnight at 4 °C. Upon incubation with the primary antibody, the specimens were washed three times in phosphate buffer solution (PBS) and visualized using 3,3′-diaminobenzidine detection kit (Dako Cytomation, Denmark). Samples were then counter stained with hematoxylin, a blue nuclear stain. Both cytoplasmic staining and membrane staining of GPRC5A and nuclear staining of p-STAT3 were considered as positive staining. To quantitate the score of GPRC5A expression, the mean percentage of positive cells was determined in at least five random fields at ×400 magnification in each section. The intensity of the immunoreactions was scored as follows: 1+, weak; 2+, moderate; and 3+, intense. The percentage of positive cells and the staining intensity then were multiplied to produce an IHC staining score [14]. P-STAT3 staining was scored as negative, 0–4%; weak, 5–29%; moderate, 30–49%; and strong, ≧50%. Strong staining was regarded as positive [15].

### Cell cultures

Human oral keratinocytes (HOK) was purchased from Chinese Beijing North, a Biotechnology Research Institute. HNSCC-derived cell lines HN12, HN13, HN30 was kindly provided by the University of Maryland School of Dentistry. The CAL27, SCC-15 and SCC-25 cell lines were purchased from the American Type Culture Collection, Manassas, VA. HOK cells were cultured in RPMI-1640 Medium (Invitrogen) supplemented with 15% fetal bovine serum (FBS). Other cells were cultured in Dulbecco’s modified Eagle’s medium (DMEM; Gibco) supplemented with 10% fetal bovine serum, 1% glutamine, and 1% penicillin–streptomycin. All cells were maintained in a humidified atmosphere of 5% CO_2_ at 37 °C.

### Plasmid construction, transfection and stable transfected cells

As described before, the coding sequence of GPRC5A was obtained from cDNA of 292G lung tumor cells and a Myc tag was added to the C-terminus using PCR. The Myc-tagged GPRC5A cDNA was then subcloned into pcDNA3.1(+) to generate the pcDNA3.1(+)-GPRC5A-Myc plasmid. The myc-taged GPRC5A plasmids and vector control plasmids were used to establish the CAL27-GPRC5A and CAL27-vector cell lines with the same methods as in our study reported previously [8].

### Western blot analysis

Different cells CAL27-vector and CAL27-GPRC5A were treated independently with IL-6 (10 ng/ml) or untreated as a control. The cells were lysed in ice-cold 2× lysis buffer containing 125 mM Tris–HCl (pH 6.8), 5% w/v SDS, and 24.75% glycerol, and subjected to total protein extraction according to standard procedure. Total 30 μg protein per lane of each sample was loaded and separated by 10% SDS–PAGE, and then electrophoretically transferred onto polyvinylidene difluoride membranes using a wet transfer system (Invitrogen). Membranes were blocked for 60 min in Tris-buffered saline (TBS, pH 7.5) with 0.5% Tween-20 (TBST) and 5% nonfat dry milk. The membranes were blocked with 5% non-fat milk powder in TBS for 1 h and probed with first antibody at 4 °C overnight. After thorough washing, the blot was incubated with secondary antibody and coupled to horseradish peroxidase-conjugated anti-rabbit (1:2000) or anti-mouse IgG (1:2000) for 1 h at room temperature. The blots were developed and the immunoreactive bands were scanned and analyzed using the Odyssey Infrared Imaging System (LI-COR Biosciences, Lincoln, NE).

### Immunofluorescence

The experimental protocol was performed as described previously [16]. Cultured cells rinsed three times with PBS and fixed with 3.7% formaldehyde were permeabilized with 0.1% Triton X-100. After blocking in 1% BSA for 1 h, cells were incubated with the primary antibody in a moist, 4 °C chamber overnight, washed and then incubated for 1 h with Alexa Fluor 488 (in the dark), or 594 donkey anti-rabbit IgG antibody (Invitrogen, Grand Island, NY) at room temperature. Washed cells (PBS containing 0.02% Tween 20), stained by mounting onto a slide with aqueous mounting medium containing 0.5 mg/ml 40–6-diamidino-2-phenylindole, were examined with a florescence microscope.

### Statistical analysis

SPSS (Statistic Package for Social Sciences) 13.0 for Windows (SPSS Inc., Chicago, IL, USA) was used to analyze data. Statistical analysis was performed using the Chi square test (for biopsies) and Student’s *t* test. When the P value was <0.05, the difference was regarded as statistically significant. The survival analysis was conducted using the Kaplan–Meier method and log-rank test.

## Results

### Patient characteristics

Of forty leukoplakia patients, twenty (50%) were men and 20 were women, with a mean age of 59 years (SD 11.6, range 32–80 years). Of 86 HNSCC patients, forty-seven were men (54.7%) and 39 were women. Thirty-three of 86 HNSCC patients (38.4%) were classified as well-differentiated, 45 (52.3%) as moderately-differentiated, and the remaining 8 (9.3%) as poorly-differentiated. The parameters of HNSCC patients in this study are presented in Table 1.

**Table 1:** GPRC5A expression and clinicopathologic features in HNSCC

Characteristic	Case number	GPRC5A staining score		Non-parametric tests value	P value
		Mean	Std. error		
Gender					
Male	47	38.12	5.56	Z = −0.213	0.831
Female	39	30.41	4.32		
Smoking history					
Yes	30	33.80	7.03	Z = −0.082	0.935
No	56	35.07	5.77		
Drinking history					
Yes	22	36.50	9.14	Z = −0.203	0.839
No	64	33.98	5.15		
Lymph node metastasis					
Yes	56	41.86	9.14	Z = −0.390	0.696
No	30	30.75	4.78		
T stage					
T1	22	20.27	3.76	χ^2^ = 0.522	0.470
T2	36	28.61	5.70		
T3	13	46.38	15.18		
T4	15	59.93	14.61		
TNM stage					
I	20	17.9	3.33	χ^2^ = 1.312	0.252
II	24	29.0	6.26		
III	21	39.9	9.67		
IV	21	51.6	12.66		
Degree of tumor differentiation					
Well	33	58.5	8.63	χ^2^ = 17.831	<0.001*
Moderately	45	22.5	4.12		
Poorly	8	3.75	1.26		

* P value <0.05 was defined as significant

### GPRC5A mRNA is frequently repressed in HNSCC

**Fig. 1:**
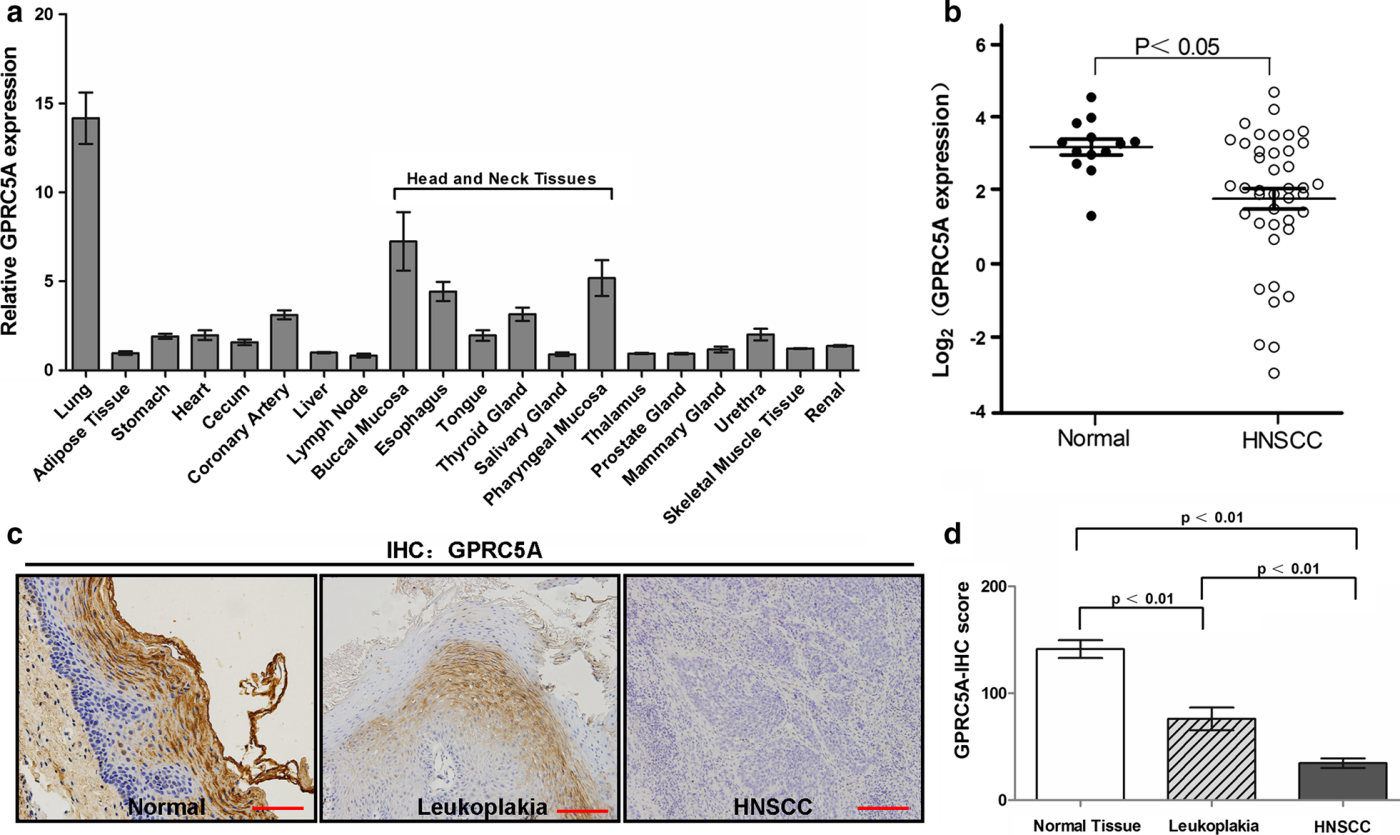
Expression pattern of GPRC5A in human normal tissues and head and neck squamous cell carcinomas. **a** Quantification of relative GPRC5A mRNA levels in many different tissues was extracted from Oncomine database. Except lung tissues, head and neck tissues shows a relative accumulation high GPRC5A expression. **b** Expression of GPRC5A were extracted from raw data published by Ginos et al. Each *circle* represents an individual sample. Whiskers (*long dashes*) in each group in both two graphs represent means, and 95% confidence intervals are represented by *short dashes*. **c** Representative images of GPRC5A expression via IHC staining in normal tissues, oral leukoplakia and HNSCC. Photos were taken under 200× magnification. **d** IHC scores of GPRC5A expression in normal tissues (n = 86), precancerous lesions (n = 40) and HNSCC (n = 86). Statistically significant differences were detected (P < 0.001)

We used public data from Oncomine (https://www.oncomine.org) for analysis of GPRC5A expression in different kinds of human organs and tissues. As previously reported, GPRC5A was expressed predominantly in lung tissues. Interestingly, GPRC5A also expressed at a relative high level in head and neck tissues compared to other organs (Fig. 1a). Moreover, analysis of GPRC5A mRNA expression by using the dataset (Ginos Head-Neck) from Oncomine showed that GPRC5A mRNA level was significantly repressed in HNSCCs as compared to that in normal tissues (P < 0.05) (Fig. 1b). Thus, expression of GPRC5A is frequently repressed in HNSCC.

### GPRC5A expression is repressed in oral leukoplakia, as well as in HNSCC

To determine which stage GPRC5A repression may occur during oncogenesis of HNSCC, we compared the expression of GPRC5A by IHC staining in 86 paired adjacent normal tissues, 40 oral leukoplakias and 86 HNSCCs. Representative IHC results for GPRC5A protein in normal tissue, precancerous lesion and primary HNSCC are shown in Fig. 1c. We found that the average IHC score of GPRC5A is high in normal tissues (141.22 ± 66.975), whereas it was significantly lower in leukoplakia (76.00 ± 67.389), and greatly repressed in HNSCC (34.63 ± 41.389) (Fig. 1d). Thus, in comparison with the expression in normal tissue, GPRC5A expression was gradually suppressed during oncogenesis of HNSCC (P < 0.01). This result suggests that repression of GPRC5A occurs at the early stage of head and neck oncogenesis, and associates with development of HNSCC.

**Fig. 2:**
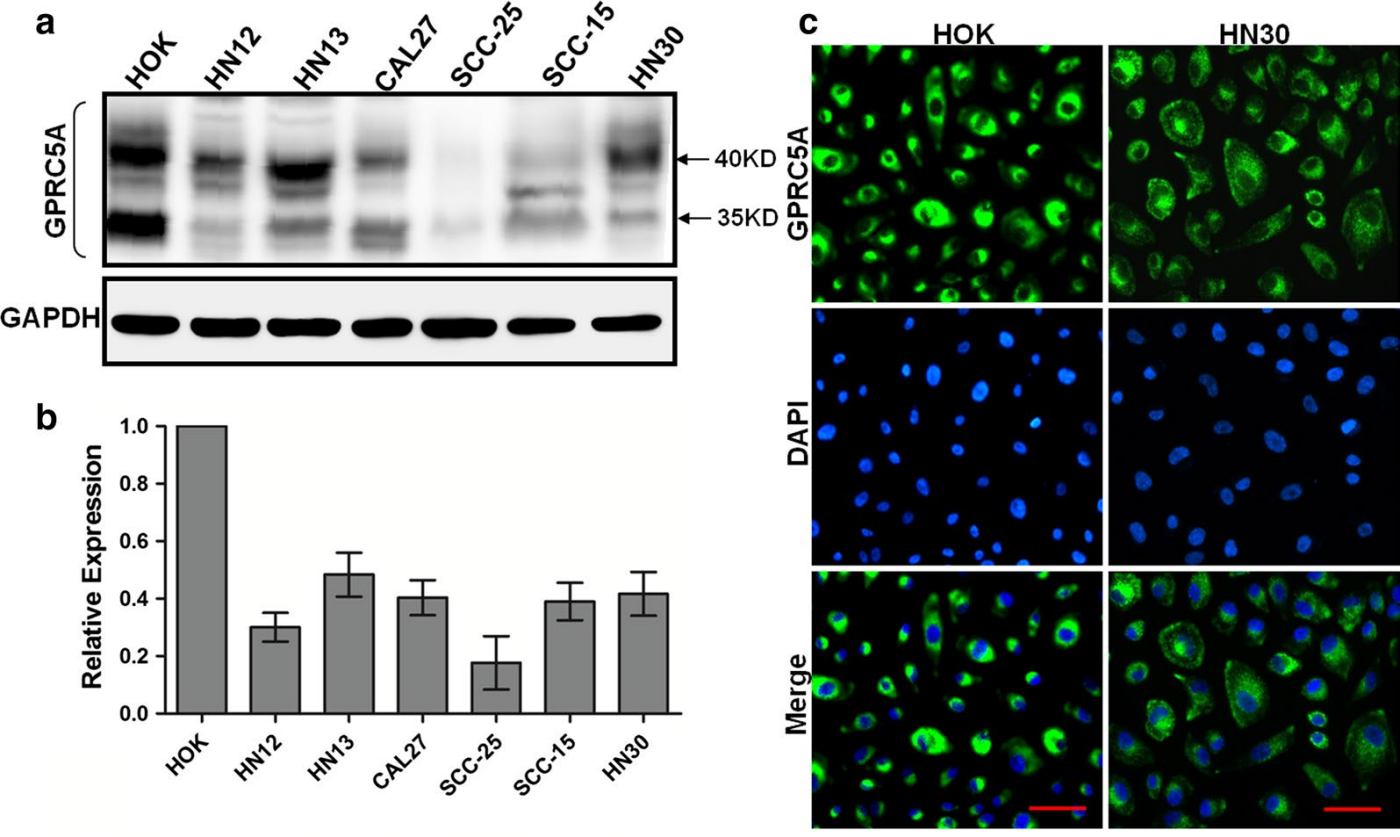
GPRC5A is frequently repressed in HNSCC cell lines. **a** Immunoblotting analysis of GPRC5A protein in the HNSCC-derived cellular lines and normal cell line HOK. GPRC5A protein expression is down-regulated in the HNSCC-derived cellular lines compared with that in the HOK. **b** Densitometric GPRC5A protein data in A are normalized to GAPDH protein levels. **c** Immunofluorescent analysis of GPRC5A subcellular localization in HOK and HN30 cells. *Bar* 50 μm

For further characterization the expression of GPRC5A in cancer cell lines, we examined the protein levels of GPRC5A in one normal cell line, HOK, and six different head and neck squamous cell lines, HN12, HN13, CAL27, SCC-25, SCC-15 and HN30, by immunoblot analysis. We found that GPRC5A protein level was the highest in HOK, but significantly decreased in several HNSCC cell lines in immunoblot (Fig. 2a). This suggests that GPRC5A expression was frequently repressed in HNSCC cells. In addition, we examined the subcellular localization of GPRC5A protein in both normal and tumor cells via immunofluorescence staining. GPRC5A was localized in the plasma membrane and intracellular vesicles, and no cellar localization difference was found between normal HOK and tumor HN30 cells (Fig. 2c). Taken together, GPRC5A was gradually repressed from normal tissue, to precancerous lesion, and to HNSCC.

### Repression of GPRC5A is associated with aggressive clinicopathological features in HNSCC patients

**Fig. 3:**
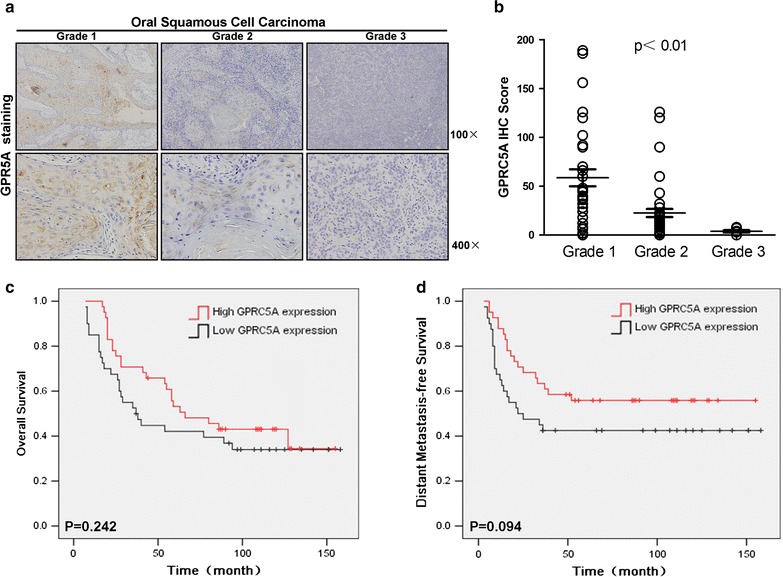
GPRC5A repression predicts aggressive clinicopathological characteristics and poor prognosis in HNSCC patients. **a** Representative images (×100 and ×400) of GPRC5A IHC staining in HNSCC with different differentiation grades. **b** IHC scores of GPRC5A expression for HNSCC of well differentiated (*Grade 1*), moderately differentiated (*Grade 2*), and poorly differentiated (*Grade 3*). **c** Low GPRC5A expression group shows a relative worse 5-year overall survival (data from Oncomine by Rickman et al.). **d** Low GPRC5A expression group shows a relative worse 5-year distant metastasis-free survival (data from Oncomine by Rickman et al.)

To further character the clinical significance of GPRC5A, we compared the clinical parameters with GPRC5A expression in 86 HNSCC patients. IHC scores of GPRC5A were analyzed based on the clinicopathological characteristics. We found that GPRC5A repression was significantly correlated with tumor grade in HNSCC tissues (P < 0.01), but was not correlated with other parameters such as gender, TNM stage, alcohol or tobacco consumption (Fig. 3a, b; Table 1). Public data which was published by Rickman et al. [14] showed that patients with lower GPRC5A expression exhibited worse 5-year overall survival and 5-year distant metastasis-free survival (Fig. 3c, d). Although the difference by Kaplan–Meier survival analysis was without significance, which was probably due to the relative small number of cases (n = 81) used, the trend is consistent with what were observed in this study, repression of GPRC5A associates with malignant phenotype of HNSCC. Taken together, these data indicate that repression of GPRC5A is correlated with tumor grade, and poor prognosis of HNSCC.

### GPRC5A repression associated with STAT3 activation, which correlates with tumor progression

**Fig. 4:**
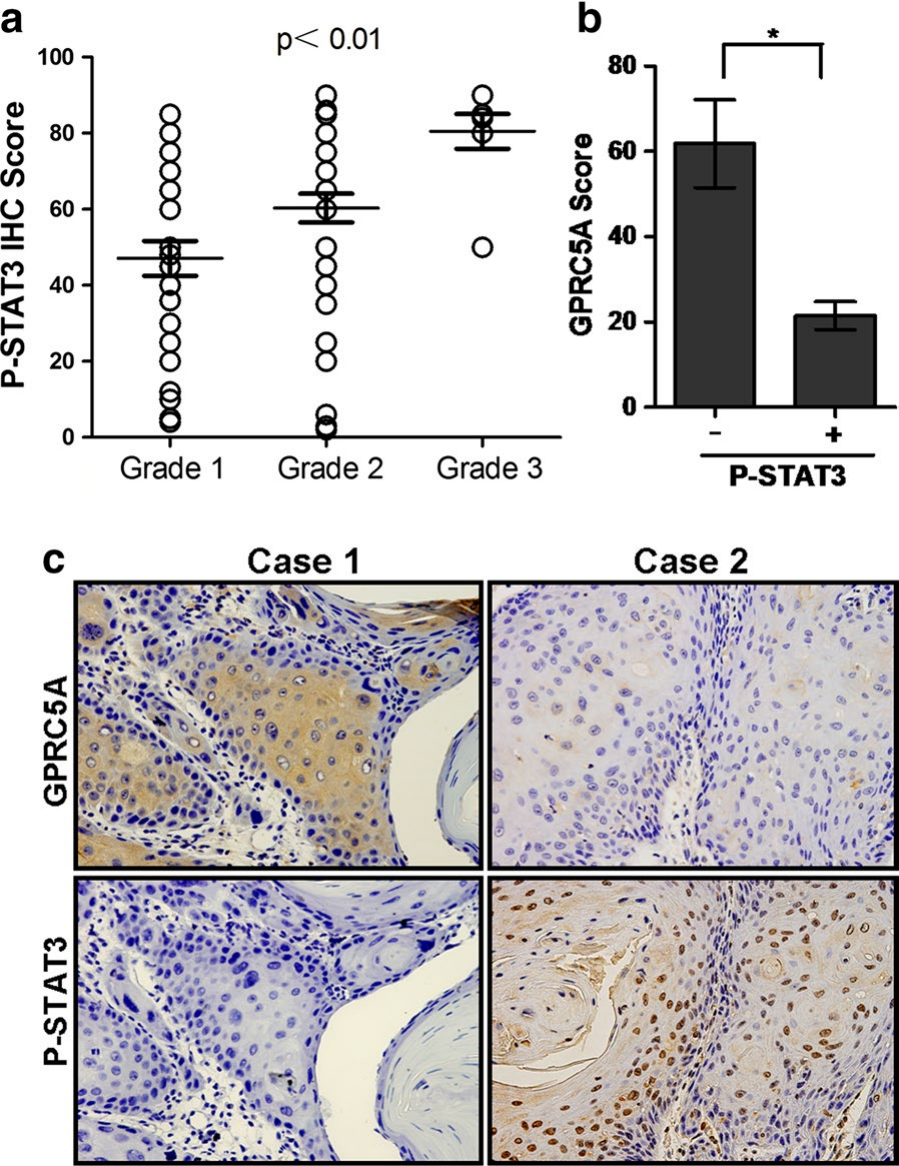
STAT3 activation, which associates with tumor progression, is inversely correlated with GPRC5A expression. **a** IHC scores of p-STAT3 expression for HNSCC of well differentiated (*Grade 1*), moderately differentiated (*Grade 2*), and poorly differentiated (*Grade 3*). **b** Statistic analysis of IHC staining for p-STAT3 in HNSCC with high and low GPRC5A (*P < 0.05). **c** Representative images (×400) of GPRC5A and P-STAT3 IHC staining in HNSCCs with different differentiation grades

To further explore the potential molecular mechanism underlying GPRC5A repression, we analyzed the genome-wide transcriptome profile of CAL27-V and CAL27-GPRC5A cells, which was described previously [8] by Agilent Whole Human Genome Microarrays. We found that inflammatory factors, especially the interleukin families such as IL-6, were reversely correlated with GPRC5A expression (data not shown). Because *Gprc5a* gene deletion has been implicated to increase STAT3 signaling in normal mouse tracheal epithelial cells (MTEC) [13], we asked if this pathway contributes to tumorigenesis of HNSCC. We then examined the activated STAT3, or p-STAT3 (Y705) in our 86 clinical HNSCC samples. IHC staining showed that the expression of p-STAT3 was correlated with tumor grade, but not other parameters of HNSCC (Fig. 4a; Table 2). Interestingly, the expression levels of GPRC5A and p-STAT3 were inversely correlated, even in different area from the same tumor tissue (Fig. 4b, c). Thus, GPRC5A repression correlates with STAT3 activation, which associates with tumorigenesis of HNSCC.

**Table 2:** P-STAT3 expression and clinicopathologic features in HNSCC

Characteristic	Case number	P-STAT3 staining score		Non-parametric tests value	P value
		Mean	Std. error		
Gender					
Male	47	53.0	8.08	Z = −1.536	0.125
Female	39	62.0	8.81		
Smoking history					
Yes	30	58.0	4.52	Z = −0.218	0.827
No	56	57.0	3.68		
Drinking history					
Yes	22	56.0	5.51	Z = −0.437	0.662
No	64	57.0	3.35		
Lymph node metastasis					
Yes	56	58.0	4.99	Z = −0.889	0.374
No	30	56.0	3.49		
T stage					
T1	22	57.0	4.28	χ^2^ = 3.011	0.390
T2	36	62.0	4.47		
T3	13	54.0	8.27		
T4	15	48.0	7.85		
TNM stage					
I	20	58.0	4.75	χ^2^ = 0.415	0.937
II	24	58.0	6.12		
III	21	60.0	5.52		
IV	21	53.0	6.34		
Degree of tumor differentiation					
Well	33	47.0	4.57	χ^2^ = 14.376	<0.01*
Moderately	45	60.0	3.76		
Poorly	8	81.0	4.55		

* P value <0.05 was defined as significant

### Overexpression of GPRC5A inhibits IL-6-induced STAT3 activation and suppresses anchorage-independent growth of HNSCC cells

**Fig. 5:**
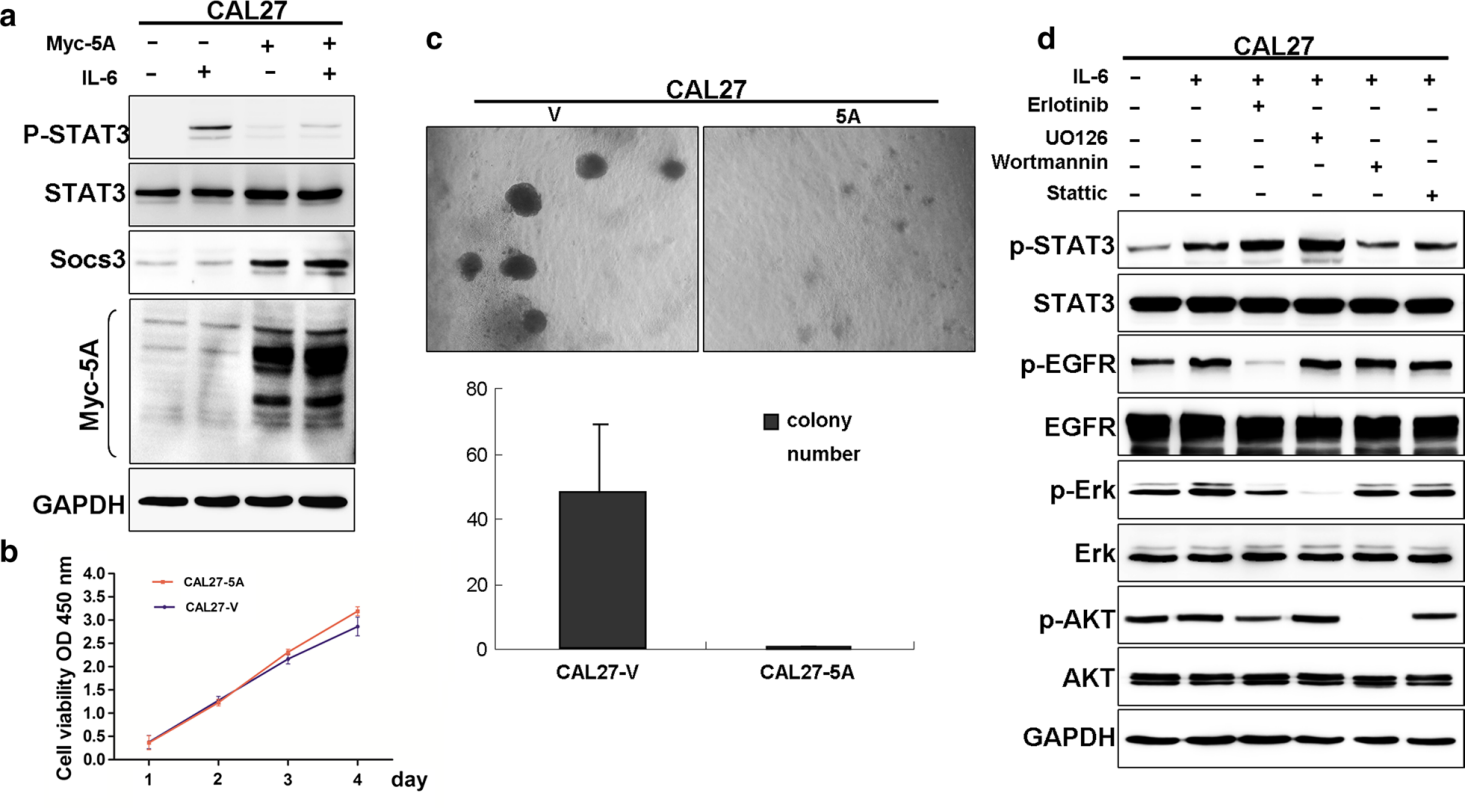
Overexpression of GPRC5A inhibits IL-6-induced STAT3 activation and suppresses anchorage-independent growth in HNSCC. **a** Immunoblot of the cell lysates from CAL27 and CAL27-GPRC5A stable transfectant cells (CAL27-5A) with the antibodies as indicated. Cells were treated with IL-6 (10 ng/ml) for 15 min. **b** The growth of CAL27 cell transfected with the pcDNA3.0 and pcDNA3.0-GPRC5A vectors were analyzed using the CCK-8 kit; the symbols represent the mean values of triplicate tests (mean ± SD). **c** Microphotographs showing colonies from anchorage-independent growth analysis of CAL27-5A and CAL27-V cells. The *bar graphs* of the mean (and standard error) number of colonies from four wells and the statistically significant differences were indicated as *asterisk* (P < 0.01) between CAL27-5A and CAL27-V cells (low) (**d**) Western blot analysis of p-STAT3, STAT3, p-EGFR, EGFR, p-AKT, AKT, P-ERK, ERK from CAL27 cells pretreated with various inhibitors for 1 h followed by stimulation with IL-6 (10 ng/ml) for 15 min

To determine the mechanism underlying the cross-talk between GPRC5A and p-STAT3, we examined the expression of STAT3 and GPRC5A in CAL27-Vector and CAL27-GPRC5A cells by treatment with IL-6. The results showed that IL-6-induced STAT3 activation was inhibited in CAL27-GPRC5A stable transfectants (Fig. 5a). Interestingly, SOCS3, a STAT3 inhibitor, is upregulated in CAL27-GPRC5A transfectants, suggesting that SOCS3 is involved in mediation of negative regulation of GPRC5A on STAT3 signaling in HNSCC cells. To determine the biological effects of GPRC5A overexpression in HNSCC, we examined the cell proliferation and anchorage-independent growth of these cells. The results showed that although cell proliferation was not changed (Fig. 5b), the colony formation was significantly suppressed in CAL27-GPRC5A cells compared to vector control (Fig. 5c). Taken together, these results suggest that overexpression of GPRC5A inhibits IL-6-induced STAT3 activation and suppress anchorage-independent growth of HNSCC cells.

STAT3 activation can be triggered by various cytokines [e.g., interleukin 6 (IL-6)] or growth factors and then regulate cell proliferation, differentiation, survival, invasion, inflammation, and immunity [11]. Previous study showed that GPRC5A deficiency leads to dysregulated STAT3 signaling via EGF-EGFR pathway in lung cancer [17]. To further determine which signaling pathway to be essential for GPRC5A-mediated inactivation of STAT3 in HNSCC cells, we examined the effects of inhibitors of EGFR, Akt, and Erk on STAT3 activation in CAL27 cells. The results showed that wortmannin, an inhibitor of PI3 K, completely blocked the activation of STAT3, whereas erlotinib and UO126, inhibitors of EGFR and Erk had no inhibitory effect on IL-6-induced STAT3 activation (Fig. 5d). This suggests that dysregulated STAT3 activation in HNSCC is independent of EGFR pathway, whereas PI3 K/Akt pathway is required.

## Discussion

In this study, we showed that GPRC5A expression was high in normal head and neck tissues, but it was gradually decreased in leukoplakia and greatly suppressed in head and neck cancer. This suggests that repression of GPRC5A occurs at an early stage and correlates with oncogenesis of HNSCC. It is noticeable that patients grouped with low GPRC5A had a worse 5-year overall survival and a worse 5-year distant metastasis-free survival than those with high GPRC5A in primary tumors. Thus, suppression of GPRC5A may serve as a molecular biomarker for the oncogenesis of HNSCC.

HNSCC is derived from epithelial origin. OSCC, which is the most common type of HNSCC, often developed in a normal–dysplasia–carcinoma sequential order. Most of OSCC undergoes through a premalignant stage. Long-term follow-up studies showed that 11–36% of oral epithelial dysplasia was transformed into OSCC [18]. Leukoplakia is the most commonly diagnosed precancerous lesion in the oral cavity [19, 20]. It has been shown that a rate of malignant transformation from leukoplakia to OSCC was between 17 and 24% with a median follow-up more than 7 years [21–23]. Thus, dysplasia has been considered the gold standard for evaluating the risk of oral cancer development [24]. Holmstrup et al. [23] stated that only non-homogeneous leukoplakias and lesions with a size exceeding 200 mm^2^ were related to a malignant transformation. However, other variables parameters examined, such as degree of epithelial dysplasia, site, smoking and surgical intervention, were not found to be statistically significant factors for malignant development. Although the follow-up data in our leukoplakia patients has not been completed, our results suggest that the status of GPRC5A expression could enhance the predictability and reliability in cancer risk assessment of oral precancerous lesions, and possibly to improve the success of treatment (Fig. 1c, d). Taken together, the results of this study support the assumption that repression of GPRC5A may play an important role in oncogenesis at premalignant stages, contributing to the progression of head and neck carcinogenesis.

Our study showed that GPRC5A was highly expressed in normal tissues, but it was down-regulated in precancerous lesion, and greatly suppressed in malignant tumors. The repression of GPRC5A was correlated with tumor grade (Fig. 3a, b). Our study is supported by the data from Oncomine in which suppressed GPRC5A has a worse 5-year overall survival and distant metastasis-free survival. Taken together, all of these results suggest that GPRC5A expression may serve as a molecular biomarker for diagnosis and prognosis of HNSCC. Development of HNSCC is associated with genetic and epigenetic changes that lead to dysregulation of cell growth and differentiation [25]. The genetic alterations are also present in premalignant lesions, suggesting a potential role in the process of malignant transformation [26–28]. The results of this study showed that GPRC5A expression is gradually repressed in oral leukoplakia and HNSCC, suggesting that repression of GPRC5A pathway is involved in the multi-step process of head and neck carcinogenesis.

GPRC5A, also known as RAI3 and RAIG-1, was originally identified as a target gene induced by all-trans-retinoic acid (ATRA). RA is known for its role as differentiation inducer [29, 30] and RA exhibits tumor suppressive activities in lung tissue. RA acts by binding to retinoic acid receptor (RAR), followed by formation of a heterodimer with retinoid X receptor (RXR). The RAR/RXR heterodimer then binds to retinoic acid response elements (RAREs) in the promoter regions of its target genes, thereby activating downstream gene transcription [31]. For example, RA stimulation induces the differentiation of ES cells into mesenchymal and skeletal muscle cells [32, 33]. The expression pattern of GPRC5A protein in normal epithelium by immunohistochemical staining is very impressive, in which GPRC5A protein exhibits at a relatively high level in those differentiated areas, but at a relative low level in those undifferentiated area (Fig. 1c). This suggests that the biological effects of GPRC5A proteins are linked to differentiation status of oral epithelium. This assumption is supported by the inverse correlation of GPRC5A expression and tumor grade of HNSCC.

An increasing number of studies have reported that STAT3 is constitutively activated in a variety of malignancies including HNSCC, and this activation is closely related to the process of carcinogenesis and tumor progression. IL-6 has been identified as the major ligand that stimulates STAT3 activation in an autocrine/paracrine fashion. STAT3 signaling, triggered by cytokines, e.g. IL-6, and growth factors, is involved in regulation of cell proliferation, differentiation, survival, invasion, inflammation, and immunity [11]. IL-6 is expressed in psoriatic skin and in cancerous epithelial cells in HNSCC, and IL-6 can be detected in the serum, tissues, and saliva of HNSCC patients [34–38]. Thus, IL-6/STAT3 seems to be a critical pathway for oncogenesis of HNSCC. In this study, we showed that p-STAT3 is correlated with tumor grade in HNSCC. Consistently, p-STAT3 expression level was high in poorly differentiated region of HNSCC, whereas STAT1 level was high in well-differentiated tumor region. It has been suggested that differentiation status of HNSCC is dependent on a STAT1/STAT3 balance [39]. In agreement with our study, all these data showed that differentiation is inversely correlated with STAT3 activation in head and neck cancer cells. In the previous study, activated STAT3 was found to be higher in *Gprc5a* ^−*/*−^ cells than in wild-type cells, suggesting GPRC5A is involved in negative regulation of p-STAT3 [13]. Moreover, repressed SOCS3 is implicated to be involved in dysregulated STAT3 activation in *Gprc5a* ^−*/*−^ MTEC. In this study, expression of GPRC5A was inversely correlated with activated STAT3, both in HNSCC cell lines and human HNSCC samples. Because GPRC5A expression is linked to inhibition of STAT3 and induction of differentiation, restoration of GPRC5A expression may provide a novel strategy for cancer prevention of HNSCC. Recently, our group reported that GPRC5A functions as a negative modulator of EGFR signaling. And repressed GPRC5A correlates with activated EGFR and STAT3 signaling in lung cancer [17], which explains dysregulated STAT3 induced by aberrantly activated EGFR in lung cancer. In this study, we found that EGFR inhibitor erlotinib did not blocked the activation of STAT3, whereas inhibitors of PI3 K/AKT showed great inhibition in HNSCC cells, suggesting that STAT3 activation in these cells is independent of EGFR pathway. The discrepancy may be dependent on the cellular context. Taken together, targeting STAT3 signaling pathway or induction of GPRC5A pathway may provide an effective strategy for prevention of oncogenesis in head and neck.

## Conclusions

In conclusion, our results demonstrate that repression of GPRC5A correlated with activation of STAT3 and contribute to the oncogenesis of HNSCC. Furthermore, the STAT3 inhibitor, SOCS3 is involved in mediation of negative regulation of GPRC5A on STAT3 signaling in HNSCC cells.

## Abbreviations

GPRC5A: G protein–coupled receptor family C group 5 member A
OSCC: oral squamous cell carcinoma
HNSCC: head and neck squamous cell carcinoma
IHC: immunohistochemical
IL-6: interleukin-6
JAK: janus kinase
HOK: human oral keratinocytes
ATRA: all-trans-retinoic acid
RAR: retinoic acid receptor
RXR: retinoid X receptor
RARE: retinoic acid response element
PBS: phosphate buffer solution
FBS: fetal bovine serum
DMEM: Dulbecco’s modified Eagle’s medium
TBS: Tris-buffered saline
